# Explainable Machine Learning for the Early Clinical Detection of Ovarian Cancer Using Contrastive Explanations

**DOI:** 10.3390/jcm14176201

**Published:** 2025-09-02

**Authors:** Zeynep Kucukakcali, Ipek Balikci Cicek, Sami Akbulut

**Affiliations:** 1 Department of Biostatistics and Medical Informatics, Inonu University Faculty of Medicine, Malatya 44280, Turkey; 2 Department of Surgery, Inonu University Faculty of Medicine, Malatya 44280, Turkey

**Keywords:** ovarian cancer, machine learning, deep learning, explainable artificial intelligence, contrastive explanation method, detection, biomarkers, diagnostic model

## Abstract

**Background**: Ovarian cancer is often diagnosed at advanced stages due to the absence of specific early symptoms, resulting in high mortality rates. This study aims to develop a robust and interpretable machine learning (ML) model for the early detection of ovarian cancer, enhancing its transparency through the use of the Contrastive Explanation Method (CEM), an advanced technique within the field of explainable artificial intelligence (XAI). **Methods**: An open-access dataset of 349 patients with ovarian cancer or benign ovarian tumors was used. To improve reliability, the dataset was augmented via bootstrap resampling. A three-layer deep neural network was trained on normalized demographic, biochemical, and tumor marker features. Model performance was measured using accuracy, sensitivity, specificity, F1-score, and the Matthews correlation coefficient. CEM was used to explain the model’s classification results, showing which factors push the model toward “Cancer” or “No Cancer” decisions. **Results**: The model achieved high diagnostic performance, with an accuracy of 95%, sensitivity of 96.2%, and specificity of 93.5%. CEM analysis identified lymphocyte count (CEM value: 1.36), red blood cell count (1.18), plateletcrit (0.036), and platelet count (0.384) as the strongest positive contributors to the “Cancer” classification, with lymphocyte count demonstrating the highest positive relevance, underscoring its critical role in cancer detection. In contrast, age (change from −0.13 to +0.23) and HE4 (change from −0.43 to −0.05) emerged as key factors in reversing classifications, requiring substantial hypothetical increases to shift classification toward the “No Cancer” class. Among benign cases, a significant reduction in RBC count emerged as the strongest determinant driving a shift in classification. Overall, CEM effectively explained both the primary features influencing the model’s classification results and the magnitude of changes necessary to alter its outputs. **Conclusions**: Using CEM with ML allowed clear and trustworthy detection of early ovarian cancer. This combined approach shows the promise of XAI in assisting clinicians in making decisions in gynecologic oncology.

## 1. Introduction

Cancer poses a significant global challenge in the 21st century, placing a significant burden on public health and the economy. It accounts for nearly one in six deaths worldwide and almost a quarter of all deaths linked to noncommunicable diseases. Moreover, cancer is responsible for about 30% of premature deaths among individuals aged 30 to 69 years and consistently ranks among the top three causes of death in this age group across the vast majority of countries [1]. Based on GLOBOCAN 2022 estimates, ovarian cancer ranks as the third most common gynecologic malignancy worldwide, following cervical and uterine cancers, with approximately 324,400 new cases and over 206,800 deaths reported annually, ranking as the 18th most commonly diagnosed cancer and the 14th leading cause of cancer death among women worldwide [1].

Ovarian cancer is one of the deadliest gynecologic cancers because it is often diagnosed only after significant progression, leading to a poorer prognosis. When the disease is confined to the ovaries (stage I), it can be cured in up to 90% of cases, and stage II is associated with a five-year survival rate of around 70%. In contrast, most cases are diagnosed at advanced stages (III or IV), where long-term survival drops to 20% or less. Currently, only about 20% of ovarian cancers are detected in early stages, emphasizing the critical need for improved screening and diagnostic strategies [2,3,4,5]. Ovarian cancer is often referred to as “the silent killer” because it usually progresses without obvious symptoms, and the lack of effective screening tools, coupled with the disease’s insidious nature, underscores the urgent need for improved early diagnostic methods and the development of reliable biomarkers [6,7,8].

Compliance with treatment protocols is a crucial determinant affecting survival rates in ovarian cancer patients. Research demonstrates that enhanced compliance with evidence-based treatment methods is associated with greater survival rates. Nonetheless, inequities persist, especially among racial and ethnic minority groups, where access to mainstream treatment is frequently restricted. High-volume cancer care centers are related with improved results, highlighting the need of concentrated, specialized treatment in efficiently managing ovarian cancer [9,10]. The incidence of ovarian cancer varies globally, similar to that of numerous other malignancies [11]. Owing to inequities in access to diagnostic and therapeutic treatments, ovarian cancer mortality exhibits a distinct pattern, with the greatest mortality rates observed in African populations [12]. The condition is believed to be influenced by various risk factors, including demographic, reproductive, gynecologic, hormonal, genetic, and lifestyle factors [13]. Although ovarian cancers are amenable to chemotherapy and often demonstrate initial responsiveness to platinum/taxane therapy, individuals with advanced disease experience 5-year recurrence rates ranging from 60% to 85% [14]. As a result, significant effort has been dedicated to the development of novel diagnostic tools to forecast the progression of the disease and the prognosis of this malignancy [15].

Machine learning (ML) and explainable artificial intelligence (XAI), which work together to provide both classification performance and interpretability, are becoming increasingly important in healthcare, particularly in the detection and diagnosis of malignant diseases, where these technologies play a crucial role in extracting meaningful patterns from large datasets [16,17,18]. For example, ML algorithms can produce more precise classification results when applied to genetic data, medical imaging, and biomarker analyses [16]. Moreover, XAI approaches foster trust in decision support systems by ensuring that the outputs of these models are interpretable for clinicians and patients. XAI and its integration with ML have thus become key areas of research, especially as artificial intelligence systems are increasingly incorporated into decision-making frameworks across various sectors. XAI seeks to enhance the comprehensibility of ML models, enabling users to understand the reasoning behind classification results and decisions. This is particularly crucial in high-stakes fields like healthcare, finance, and autonomous systems, where understanding model behavior can profoundly influence outcomes and trust in AI technologies. The need for explainability arises from the inherent complexity of many ML models, especially deep learning architectures, which often function as “black boxes,” offering minimal transparency about their internal processes. Kobylińska et al. [19] have highlighted that explainable ML techniques are increasingly applied in areas like lung cancer screening, where understanding model classification decisions can improve clinical outcomes. Similarly, Allen’s research [20] on county-level obesity incidence demonstrates how interpretable models can outperform traditional epidemiological approaches, revealing critical determinants of health outcomes. The expanding body of XAI research also explores the technical methodologies for achieving explainability. Roscher et al. [21] have discussed how XAI contributes to scientific understanding by clarifying the decision-making processes of ML models. Techniques such as feature importance scores, counterfactual explanations, and decision trees are frequently employed to elucidate model behavior and enhance user comprehension [22].

The Contrastive Explanation Method (CEM) is an XAI technique that enhances the interpretability of ML models by revealing not only the reasons behind a specific classification decision (the evidence supporting the classification result) but also the minimal changes required to alter that decision to a different class. CEM provides two types of explanations: pertinent positives (PP), which emphasize features that strongly justify the current classification, and pertinent negatives (PN), which describe the adjustments needed to achieve an alternative outcome. This technique is especially valuable in healthcare, as it enables clinicians to grasp both the rationale for a model’s classification decision and the conditions under which that decision might change [16,23,24].

Given that ovarian cancer often remains asymptomatic during its early stages and is typically diagnosed at advanced stages, this study seeks to develop an early detection strategy by integrating XAI and ML. Accordingly, an open-access dataset containing data from patients with ovarian cancer and benign ovarian tumors is used to train an ML classification model, which is further explained using the CEM to clarify the model’s decision-making process.

## 2. Material and Methods

### 2.1. Data Collection and Variables

The patient cohort utilized in the current study comprised individuals diagnosed with ovarian cancer and benign ovarian tumors. All patients included in the study underwent surgical resection, and their diagnoses were confirmed through detailed pathological examination post-surgery. Importantly, none of the ovarian cancer patients received any form of chemotherapy or radiotherapy prior to their surgical procedures, ensuring that the data reflects the untreated state of the disease.

The dataset encompasses a total of 349 patients with ovarian cancer, all of Chinese descent. This dataset is rich in variables, featuring 49 distinct parameters that include demographic information, results from blood routine tests, general biochemical data, and tumor marker levels. These variables were meticulously collected to provide a multidimensional view of patient health and clinical status [15]. In this study, the publicly available dataset was expanded twofold using the bootstrap resampling method in order to increase the sample size and enhance the statistical power of the analyses. Notably, this resampling procedure was applied solely for the development and internal validation of machine learning models. It was not used for descriptive statistics or comparative analyses, which were conducted exclusively on the original dataset to preserve the authenticity of clinical and demographic characteristics. By repeatedly and randomly sampling with replacement from the original data, an augmented dataset was generated and subsequently utilized during the modeling process.

The target variable for the analysis was the classification of patients into one of two categories: ovarian cancer or benign ovarian tumor. This distinction was critical for developing detection models and identifying potential biomarkers for diagnosis. Such a dataset holds significant promise for improving diagnostic accuracy and enhancing our understanding of the factors differentiating malignant from benign ovarian conditions.

### 2.2. Statistical Analysis and Feature Selection

In the data analysis process, Lasso (Least Absolute Shrinkage and Selection Operator) regression was employed to identify the optimal set of variables capable of successfully detecting ovarian cancer. Lasso is an effective feature selection method that improves the generalization capability of models by reducing the number of variables in complex models through L1 regularization technique. This method offers the advantage of enhancing detection performance while reducing model complexity, particularly in high-dimensional datasets.

The feature selection process using Lasso regression was conducted through the following steps:

1. All biomarkers, demographic data, and laboratory parameters were evaluated in the initial model.
2. The optimal regularization parameter (lambda) was determined using 10-fold cross-validation.
3. Lasso regression was applied with the determined lambda value, and the variables that contributed most to the model [Age, Alpha-fetoprotein (AFP), Albumin (ALB), Alkaline phosphatase (ALP), Carbohydrate antigen 125 (CA125), Carcinoembryonic antigen (CEA), Human Epididymis Protein 4 (HE4), lymphocyte count (LYM), plateletcrit (PCT), platelet (PLT), Red blood cell (RBC), and Total bilirubin (TBil)] were selected.
4. The selected variables were used in the training of the deep learning model.

Statistical group comparisons were performed using SPSS (version 25.0) software. For comparisons between cancer and non-cancer groups, the Mann–Whitney U test was used as the data did not meet the assumptions for parametric testing. A *p*-value < 0.05 was considered statistically significant in all analyses.

The ML modeling and CEM analyses were implemented in Python 3.10. The model’s performance was evaluated using metrics such as accuracy, sensitivity, specificity, F1-score, and the Matthews correlation coefficient (MCC). The 95% confidence intervals for these metrics were calculated using the bootstrap method to test the reliability of the model.

### 2.3. Deep Learning Model and Training Process

The deep learning model developed in the study has a three-layer feedforward neural network architecture. The model consists of an input layer with 12 neurons, two hidden layers with 64 and 32 neurons respectively, and an output layer with 2 neurons. ReLU activation function was used in the hidden layers, and softmax activation function was used in the output layer.

Model training was performed using the Adam optimization algorithm and categorical cross-entropy loss function. During the training process, batch size was set to 32, and the model was trained for up to 100 epochs. The data was subjected to normalization and then divided into training (80%) and test (20%) sets. We applied Z-score standardization using sklearn’s StandardScaler, where scaling parameters (mean μ and standard deviation σ) were fitted exclusively on the training dataset. These training-derived parameters were then applied to both validation and test sets, ensuring no information leakage from unseen data. Specifically, the scaling formula z = (x − μ_train)/σ_train was applied consistently across all data splits. Post-scaling verification confirmed that the training set achieved the expected mean ≈ 0 and standard deviation ≈ 1, while test set statistics appropriately differed from these values, as expected when using training-fitted parameters. Missing values were handled by median imputation.

The classification model was developed using TensorFlow and Keras libraries. We used the Alibi library for model explainability and to generate explanations of detection decisions. Data analysis and visualization were performed using Pandas 2.3.1, NumPy 1.26.4, Scikit-learn 1.7.0, Seaborn 0.13.2, and Matplotlib libraries 3.10.3.

To ensure reproducibility and enhance model robustness, several training strategies and hyperparameter settings were applied during the development of the deep neural network. Dropout regularization was set to 0.3 for both hidden layers to reduce overfitting. Early stopping was implemented with a patience of 15 epochs, monitoring the validation loss and restoring the best-performing weights via restore_best_weights = True. Additionally, PyTorch v1.12.0’s learning rate scheduler (ReduceLROnPlateau) was used, with a reduction factor of 0.5 and a patience of 8 epochs, allowing the learning rate to adapt when validation performance plateaued.The network was trained using the Adam optimizer with its default parameters: learning rate = 0.001, β_1_ = 0.9, β_2_ = 0.999, and ε = 1 × 10^−7^. These configurations were selected through empirical tuning and align with standard practices in deep learning, ensuring both convergence and generalizability. The trained model was then evaluated on an independent test set to measure its generalization capability on real-world data.

### 2.4. The Contrastive Explanation Method (CEM)

XAI has emerged as a pivotal area of research aimed at enhancing the interpretability and transparency of artificial intelligence systems. The complexity of modern ML models, particularly deep learning architectures, often results in a “black box” phenomenon where the decision-making processes are obscured from users. This lack of transparency can hinder trust and reliability in artificial intelligence applications, especially in high-stakes domains such as healthcare, finance, and law [25,26]. To address these challenges, XAI seeks to develop methodologies that elucidate how AI systems arrive at their conclusions, thereby fostering user trust and facilitating better decision-making [27].

One prominent method within the XAI framework is the CEM, which focuses on providing explanations that highlight the differences between what happened and what could have happened under different circumstances. The CEM is an emerging technique within the realm of XAI that aims to enhance the interpretability of ML models, particularly black-box models like deep neural networks (DNNs). CEM operates by providing explanations that contrast the detection outcome with alternative scenarios, effectively answering the question, “Why did the model detect this case as Cancer instead of No Cancer?” This method is particularly valuable in domains such as healthcare, where understanding the rationale behind detection decisions is crucial for clinical decision-making [28]. The ability of CEM to generate contrastive explanations allows it to elucidate the decision-making process of ML models by comparing actual outcomes with hypothetical alternatives, thereby enhancing user trust and understanding [29].

The theoretical foundation of CEM is rooted in counterfactual reasoning, which posits that explanations are more comprehensible when they are framed in terms of what could have happened under different circumstances. This aligns with findings that suggest contrastive explanations can be cognitively less demanding for users, as they provide a clearer context for understanding model behavior [30]. Furthermore, CEM has been shown to complement other interpretability techniques, such as Shapley values, by offering a more intuitive grasp of model predictions through relatable scenarios [31]. In practical applications, CEM has demonstrated its utility in various fields, particularly in healthcare, where it aids in making complex detection decisions more interpretable for clinicians and patients alike [32]. The method’s adaptability to different datasets and its capacity to produce meaningful descriptions make it a valuable tool in the ongoing quest for transparency in AI systems. In summary, the Contrastive Explanation Method stands out as a significant advancement in the field of XAI, providing a structured approach to generating explanations that enhance the interpretability of complex ML models. Its reliance on counterfactual reasoning and its application across various domains underscore its importance in fostering trust and understanding in AI-driven detection processes [28].

## 3. Results

### 3.1. Demographic and Descriptive Characteristics

The study’s data set comprised 349 patients, including 171 with ovarian cancer and 178 with benign ovarian tumors. The average age of the patients was 45.05 ± 15.13. The average age of patients with ovarian cancer was 52.98 ± 13.48 years, whereas the average age of patients with benign ovarian tumors was 37.44 ± 12.5 years.

### 3.2. Descriptive Statistics of Biomarkers and Comparison Between Groups

The median values of biomarkers for the comparisons between groups are presented in Table 1. Statistically significant differences were detected between cancer and non-cancer patients for all biomarkers (*p* < 0.05). ALB (43.4 vs. 39.2), LYM (1.67 vs. 1.34), RBC (4.42 vs. 4.32), and TBIL (9.2 vs. 7.6) values were found to be significantly higher in cancer patients compared to non-cancer patients. On the other hand, AFP (2.1 vs. 2.44), ALP (65 vs. 77), CA125 (22.66 vs. 241.5), CEA (1.27 vs. 1.41), HE4 (43.77 vs. 140.9), PCT (0.23 vs. 0.26), and PLT (223.5 vs. 265) values were significantly lower in cancer patients compared to non-cancer patients.

### 3.3. Model Performance Evaluation

**Figure 1:**
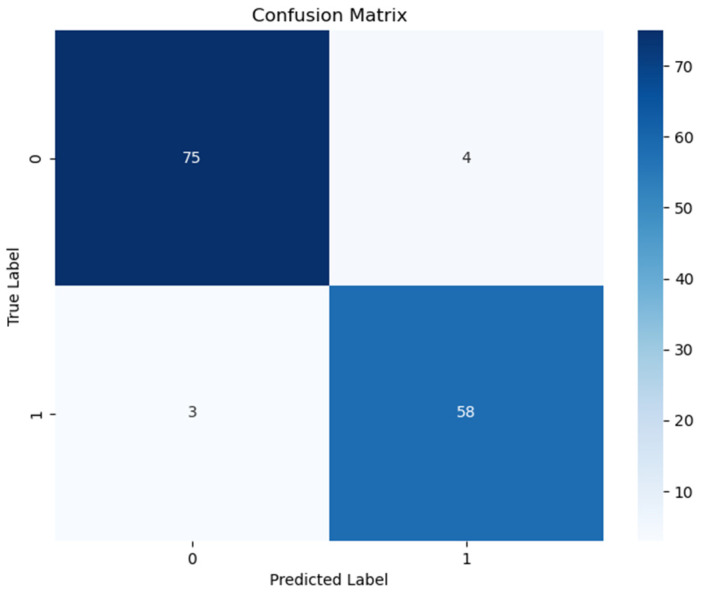
Confusion matrix for the model.

The performance metrics of the developed model on the test set are shown in Table 2. The model demonstrated superior performance in cancer classification, achieving high accuracy, sensitivity, specificity, and F1-score values. In addition, the optimal classification thresholds used for generating these metrics are also reported in Table 3 to ensure full transparency and reproducibility of the model evaluation. The confusion matrix for the model is shown in Figure 1.

According to Table 2, the deep neural network model demonstrated high overall performance on the test set. The model achieved strong sensitivity (0.962) and specificity (0.935), with high predictive values [positive predictive value (PPV): 0.949; negative predictive value (NPV): 0.951], indicating reliable discrimination between cancer and non-cancer cases. The F1-score (0.955), balanced accuracy (0.949), and Matthews Correlation Coefficient (0.899) further support the model’s robustness. Additionally, assessed on the observed class prevalence of 0.530, the model achieved a PPV of 0.949 and an NPV of 0.951. These high predictive values indicate that the model maintains a strong ability to correctly identify both diseased and non-diseased individuals, which is critical for clinical applicability. The inclusion of class prevalence in the calculation ensures that these values reflect real-world diagnostic performance more accurately.

To assess the model’s behavior under different classification thresholds, we evaluated key performance metrics across a range of clinically meaningful threshold values. According to Table 3, the model achieved consistently high performance across all thresholds. The table summarizes model performance at thresholds of 0.1, 0.3, 0.5, 0.7, and 0.9, each corresponding to distinct clinical priorities (e.g., maximizing sensitivity vs. specificity). The threshold of 0.1, identified as optimal by Youden’s J index, yielded the highest overall balance (Youden = 0.917) with excellent sensitivity (0.967) and NPV (0.974), making it suitable for early detection scenarios where minimizing false negatives is critical. In contrast, higher thresholds (e.g., 0.7 and 0.9) increased PPV and specificity, which may be more appropriate in confirmatory or resource-limited settings where reducing false positives is a priority. These results demonstrate the model’s adaptability to different clinical decision-making contexts.

**Figure 2:**
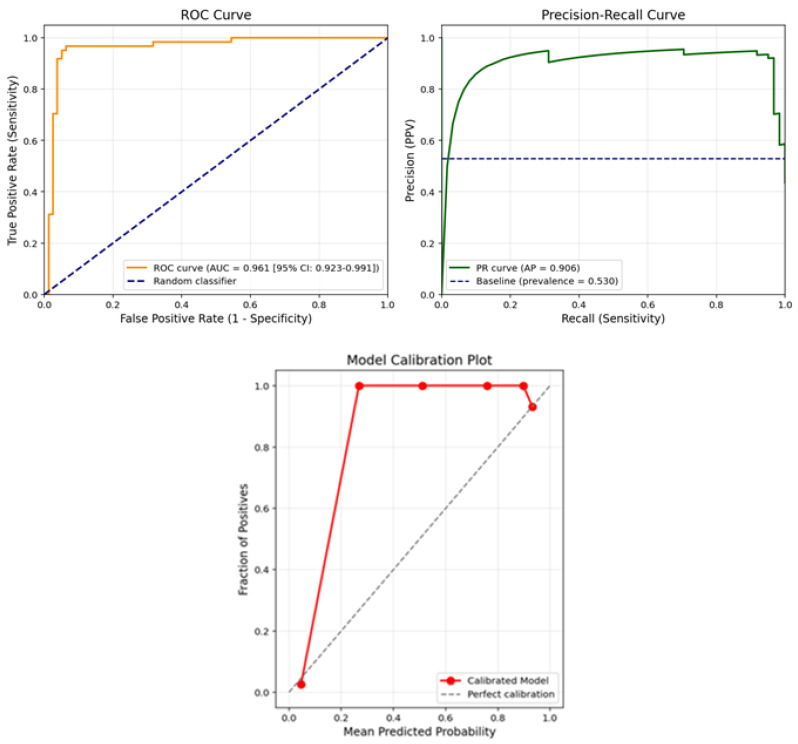
The ROC, PR curve and calibration plot for the model.

The ROC, PR curve and calibration plot for the model are shown in Figure 2.

Figure 2 presents the receiver operating characteristic (ROC) curve and the calibration plot of the final model on the test set. As shown in the ROC curve (left panel), the model achieved an area under the curve (AUC) of 0.961 with a 95% confidence interval of 0.923–0.991, indicating excellent discriminative ability in distinguishing ovarian cancer cases from benign tumors. The curve remains well above the diagonal baseline (random classifier), demonstrating high sensitivity across a wide range of specificity values.

In addition, the model’s reliability was assessed using a calibration plot (right panel), which compares the predicted probabilities against the actual observed outcomes. The plot shows that the predicted probabilities align closely with the true outcome frequencies, especially in higher probability bins. The model slightly overestimates risk near the upper range but maintains strong agreement with the diagonal line of perfect calibration across most probability thresholds.

Together, these plots confirm that the proposed deep learning model not only classifies cases with high accuracy but also provides well-calibrated probability estimates, which is essential for clinical decision-making and risk stratification in ovarian cancer diagnosis.

### 3.4. Explainable Artificial Intelligence Method (CEM) Results

The CEM was used to enhance the interpretability of the model. CEM analysis was performed in two different modes: pertinent positive (PP) and pertinent negative (PN). Model results on two example patients were examined. In these analyses, kappa value was set to 0.2, beta value to 0.1, and a maximum of 1000 iterations were used. In the CEM analysis, we set the kappa parameter to 0.2 to ensure a minimum confidence margin for counterfactual classification, and beta to 0.1 to promote sparse explanations by penalizing changes across too many features. These values were selected based on prior work and empirical tuning to balance interpretability and model faithfulness.

#### 3.4.1. Example Patient Analysis: Explainability Investigation in a Patient Diagnosed with Cancer

As part of the CEM conducted to evaluate the decision mechanism of the model, a patient diagnosed with cancer was analyzed. This patient was classified as “Cancer” by the deep learning model with a probability of 99.68%. The standardized feature vector for this patient includes a total of 12 parameters: AFP, Age, ALB, ALP, CA125, CEA, HE4, LYM, PCT, PLT, RBC, and TBIL.

##### Pertinent Positive (PP) Analysis

PP analysis shows the most determining biomarkers in the model’s assignment of the patient to the “Cancer” class. When evaluating the PP vector of this patient, particularly LYM (1.357), RBC (1.179), PLT (0.384), and PCT (0.036) parameters provided high positive contributions. These findings indicate that hematological indicators such as LYM, RBC, PLT, and PCT play a distinctive role in cancer diagnosis.

##### Pertinent Negative (PN) Analysis

PN analysis reveals the minimum biomarker changes required for the classification to be reversed (i.e., transition from “Cancer” class to “No Cancer” class). According to the analysis results, significant increases in the age parameter from −0.133 to 0.233 and in HE4 value from −0.427 to −0.050 stand out as critical determinants for class change. Changes in other parameters were minimal and did not have a significant effect on classification.

##### Clinical Comment

The CEM analysis on this example patient provides important information in explaining the decision mechanism of the model. It has been observed that small but strategic changes in Age and HE4 parameters could enable the transition from Cancer class to No Cancer class. On the other hand, it was determined that the positive contributions supporting the current classification largely derive from parameters such as LYM, RBC, PLT, and PCT. These results reveal that not only individual biomarker levels but also interactions between biomarkers should be considered in the diagnostic process.

**Figure 3:**
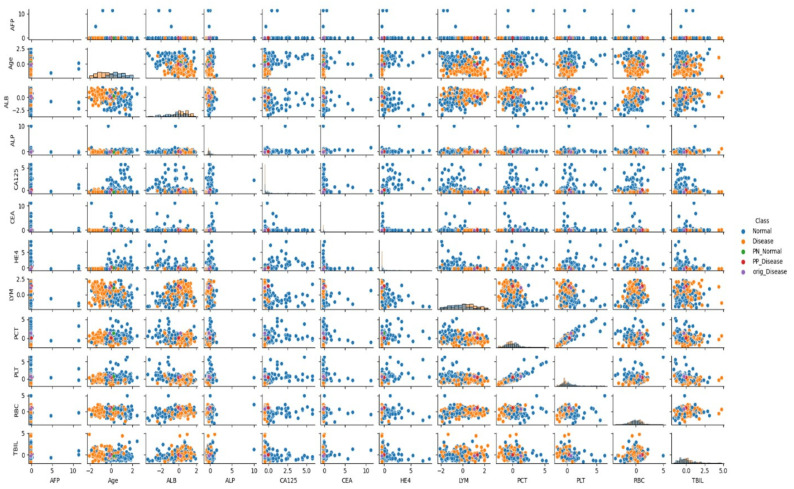
Scatter Matrix Depicting Biomarker Relationships in Patients Diagnosed with Ovarian Cancer.

The specific biomarker values of the analyzed patient, along with the impact of changes in these values on classification and the key positive and negative determinants of the model’s decision, are detailed in Table 4 and Figure 3.

Figure 3 visually presents the pairwise relationships among the 12 selected biomarkers using a scatter matrix, overlaid with class labels (Normal vs. Disease) and CEM-based PP/PN indicators. This figure specifically focuses on patients diagnosed with ovarian cancer, allowing a detailed visualization of biomarker behavior within this clinically significant subgroup. The distribution patterns reveal clear separation between cancer and non-cancer cases in key feature combinations, particularly HE4–CA125, RBC–PCT, and LYM–ALB.

The inclusion of PP and PN vectors enhances the clinical interpretability of the model by illustrating which features contribute to or require changes for class transitions. Notably, PN points are positioned at the distribution edges, consistent with the CEM findings that identified substantial increases in Age and HE4 or decreases in RBC as critical for class reclassification. Overall, this figure reinforces the alignment between model-based contrastive explanations and biomarker dynamics observed in ovarian cancer patients, strengthening the clinical interpretability of the proposed approach.

#### 3.4.2. Example Patient Analysis: Explainability Investigation in a Patient Not Diagnosed with Cancer

The second example of explainability analyses using the CEM was conducted on a patient classified as “No Cancer” by the model with a probability of 99.74%. The standardized biomarker vector for this patient includes the parameters AFP, Age, ALB, ALP, CA125, CEA, HE4, LYM, PCT, PLT, RBC, and TBIL.

##### Pertinent Positive (PP) Analysis

PP analysis evaluates the contribution of biomarkers that keep the patient in the “No Cancer” class. According to the analysis findings, the PP values of all biomarkers were found to be close to zero, indicating that no single biomarker was dominant in the classification decision. Instead, it is thought that the biomarker combination formed by values such as ALB, ALP, PCT, and LYM collectively contributes to keeping the patient in the “No Cancer” class.

##### Pertinent Negative (PN) Analysis

PN analysis shows which biomarkers need to change for the patient’s current class to be reversed and classified as “Cancer”. As a result of this analysis, the most critical change was determined to be in the erythrocyte count (RBC) parameter. A decline in RBC value from 0.08 to −0.96 is the most significant biomarker change required for the model to change its class decision. Additionally, decreases observed in AFP, ALP, CEA, LYM, and PLT parameters were also found to contribute to the class transition. These findings suggest that a decrease in hematological and tumor-related markers could trigger a cancer diagnosis.

##### Clinical Comment

The CEM analysis of this patient shows that in the classification of individuals without cancer, the model relies on patterns across multiple biomarkers rather than any single dominant biomarker. In particular, decreases in hematological and tumor markers such as RBC, LYM, PLT, and CEA are critical changes that could cause the patient to transition to the Cancer class. However, the absence of any dominant positive marker supporting the current classification reveals that the model uses a more distributed and collective pattern when defining the No Cancer class. This indicates the importance of evaluating not only individual markers but also biomarker profiles together in the development of clinical decision support systems.

**Figure 4:**
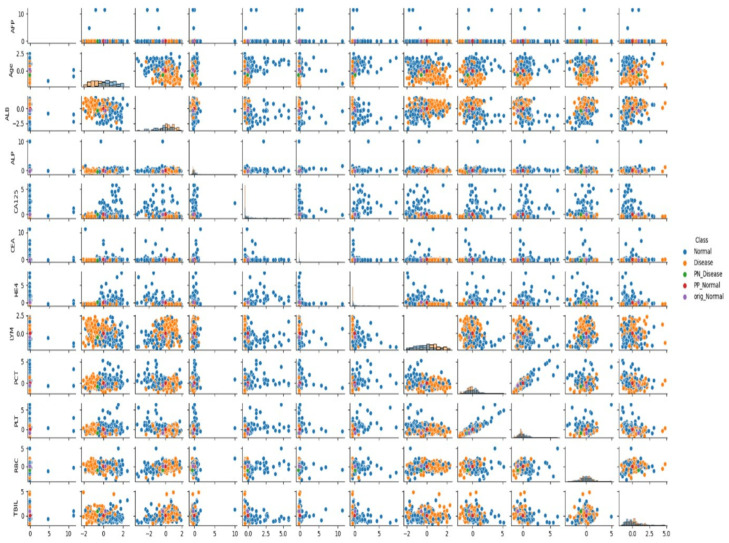
Scatter Matrix Depicting Biomarker Profiles in Patients Without Ovarian Cancer.

The specific biomarker values of the analyzed non-cancer patient, along with the impact of changes in these values on classification and the key positive and negative determinants of the model’s decision, are detailed in Table 5 and Figure 4.

Figure 4 illustrates the pairwise relationships among the 12 selected biomarkers in patients without ovarian cancer, using a scatter matrix format. The visualization includes class labels (Normal vs. Disease) and CEM-derived PP and PN points, focusing specifically on the “No Cancer” classification context.

Distribution patterns reveal distinct clustering between Normal and Disease cases in certain biomarker combinations—particularly RBC–PLT, LYM–ALB, and CA125–HE4—highlighting the discriminatory power of these variables even in non-cancer populations. Notably, PN points (representing features that would shift a “No Cancer” classification to “Cancer”) often appear at the lower bounds of RBC or higher ranges of CA125 and Age, consistent with the biologically plausible changes identified by the CEM algorithm. The figure also demonstrates strong alignment between the model’s contrastive explanations.

Overall, Figure 4 serves as a complementary visualization to Figure 3 illustrating how distinct patterns of biomarker variation underpin the model’s classification decisions in the absence of ovarian cancer, while validating the relevance of contrastive features identified through CEM.

### 3.5. Analysis of Feature Relationships

In the presented visuals, patients diagnosed with cancer are labeled as “Cancer” (orange dots), and patients without cancer diagnosis are labeled as “No Cancer” (blue dots). The visuals also include labels such as “PN_No Cancer”, “PP_No Cancer”, “PN_Cancer”, and “PP_Cancer”, which show the PP and PN vectors used in the CEM analysis. These visuals help us understand which biomarker changes affect classification.

As can be seen in Figure 1 and Figure 2, when the relationships between biomarkers are analyzed, it has been determined that certain parameters show strong interactions with each other in cancer classification. Particularly, relationships between AFP, ALB, CA125, and RBC, LYM, PCT, and TBIL values play an important role in distinguishing between cancer and non-cancer cases.

When examining the data distribution, it was observed that cancer and non-cancer cases are distinctly separated in some biomarker combinations. Especially the CA125-HE4 relationship and ALB-LYM relationship show clear distinctions between classes. These relationships play an important role in the model’s classification decisions.

### 3.6. Clinical Importance of CEM Analysis

CEM analysis transparently reveals which laboratory parameters the ML model uses for classification. As a result of two example patient analyses:

1. It was shown that LYM, RBC, PLT, and PCT parameters have a determining role in the diagnosis of cancer patients,
2. A holistic evaluation of laboratory parameters is important in non-cancer individuals,
3. Age and HE4 values require critical parameter changes for transition from Cancer class to No Cancer class,
4. In non-cancer patients, the decrease in RBC value is the most determining factor in the transition to the Cancer class,
5. Evaluating biomarkers not alone but in interaction with each other gives more accurate results.

These findings are also supported by the distributions seen in Figure 1 and Figure 2, and provide important clues for the development of clinical decision support systems in cancer diagnosis.

### 3.7. Clinical Contribution of XAI and Contrastive Explanations

The explainable artificial intelligence method and Contrastive Explanations used in this study provide the following advantages to clinicians, in addition to providing high-accuracy classification in the cancer diagnosis process:

1. Understanding and confidently applying the recommendations behind the artificial intelligence model
2. Guidance on which biomarker changes may affect patients’ health status
3. Determination of critical biomarkers for early diagnosis and monitoring of the disease
4. More accurate diagnosis by effectively applying multivariate analysis

Contrastive Explanations make patient management safer and more effective by showing clinicians not only the current situation but also potential scenarios and intervention points.

### 3.8. Model Comparisons

This section compares the performance metrics of lasso logistic regression, XGBoost, and the Deep Neural Network model we applied using the data set obtained through variable selection. The performance metrics table for the models is presented in Table 6. The variable importance scores obtained from Lasso logistic regression and XGBoost are also presented in Table 7.

When the results of the CEM analysis were examined, age and HE4 were found to be critical variables within the cancer group. The results obtained from the two modeling studies support these findings.

### 3.9. Deep Neural Network and SHAP Analysis

**Figure 5:**
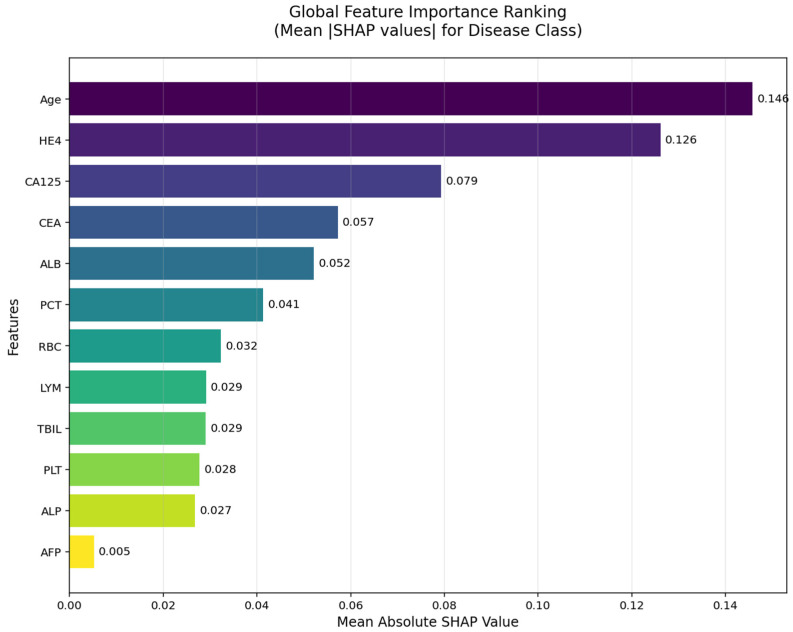
Global Feature Importance Ranking Based on SHAP Values (Darker shades (purple) correspond to features with higher importance, Lighter shades (yellow/green) represent features with lower importance).

**Figure 6:**
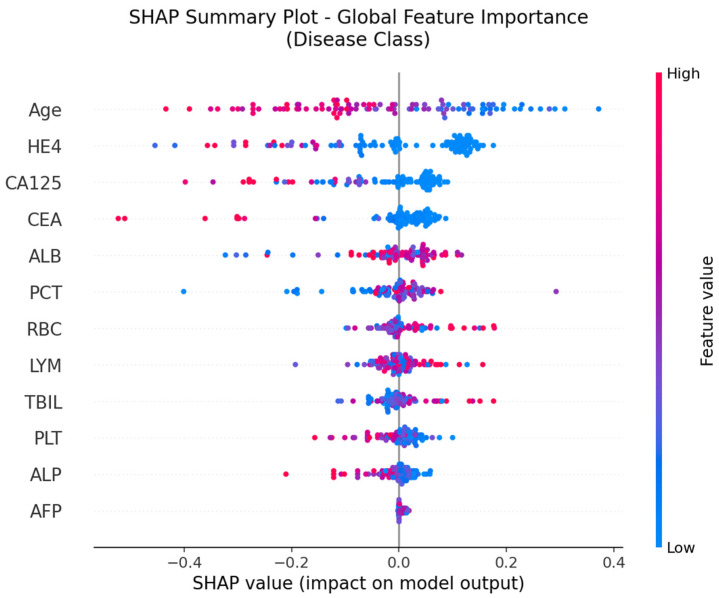
SHAP Summary Plot.

The SHAP global feature importance graph and SHAP summary graphs obtained by applying the SHAP architecture to the deep neural network model used in the CEM analysis are shown in Figure 5 and Figure 6.

According to the SHAP-based analyses presented in Figure 5 and Figure 6, several key biomarkers were identified as having substantial influence on the model’s prediction of ovarian cancer. In the global importance ranking (Figure 5), Age, HE4, and CA125 emerged as the top three predictors, indicating that both tumor markers and clinical-demographic variables play a prominent role in the model’s decision-making process. These findings are consistent with clinical expectations and prior literature, as advanced age and elevated tumor marker levels are well-known indicators of malignancy in ovarian pathology.

Figure 6, which illustrates the SHAP summary plot, provides a more detailed view of how individual values of each feature contribute to the model’s output. Notably, high values of Age, HE4, and CA125 (shown in red) tend to push predictions toward the “Cancer” class, while higher values of ALB, LYM, RBC, and TBIL are generally associated with a decreased cancer probability. This directional insight aligns with the model’s learned patterns and also corroborates the CEM analysis, in which lymphocyte count (LYM), RBC, and PLT were identified as positive contributors in cancer cases, and Age and HE4 were critical variables in class transition scenarios.

## 4. Discussion

Ovarian cancer remains the most lethal form of gynecological malignancy, primarily due to the difficulty in detecting the disease at an early stage and its high rate of recurrence following treatment [33,34]. One of the most significant challenges in managing ovarian cancer is the lack of reliable and sensitive screening tools for early diagnosis. As a result, the majority of patients are diagnosed when the disease has already progressed to an advanced stage, typically stages III or IV, by which point the tumor burden is substantial, and therapeutic options are less effective [35]. This late-stage diagnosis considerably worsens the clinical outcome, often leading to poor prognosis and increased mortality.

Statistical data further emphasize the critical importance of early detection. Patients diagnosed at an early stage (FIGO stages I and II) exhibit a markedly higher 5-year survival rate, exceeding 90%. In contrast, those diagnosed at advanced stages (III and IV) have significantly poorer outcomes, with 5-year survival rates dropping to approximately 20–40%. Despite advances in surgical techniques, chemotherapy regimens, and targeted therapies, the overall 5-year survival rate for ovarian cancer across all stages remains approximately 45%, positioning it as the second deadliest gynecologic cancer after uterine cancer [36,37,38].

Given these alarming survival statistics and the tendency for ovarian cancer to present with nonspecific or no symptoms in its early stages, there is a pressing need to develop and implement more effective diagnostic strategies. These strategies should focus particularly on the identification and validation of novel molecular and clinical biomarkers that can aid in the detection of ovarian cancer at an earlier, more treatable stage. Studies aimed at elucidating such early diagnostic markers are of paramount importance, as they have the potential to not only facilitate timely diagnosis but also guide personalized treatment approaches. This could significantly reduce disease-related morbidity and mortality by intervening before the cancer progresses to an advanced stage with limited therapeutic options.

The CEM is a state-of-the-art XAI technique designed to improve the interpretability of complex ML models, particularly those with “black box” architectures such as DNNs. In this study, we utilized CEM as a core component of our diagnostic modeling framework due to its ability to provide Contrastive Explanations, which highlight the minimal changes required to alter a model’s prediction. This approach is especially valuable in clinical contexts like ovarian cancer diagnosis, where understanding why a patient was classified as “Cancer” rather than “No Cancer” (and vice versa) can provide actionable insights to clinicians [39].

The rationale behind selecting CEM stems from the critical clinical need to enhance transparency and trust in AI-driven decision-support systems. Unlike traditional feature importance metrics that only reveal which variables influenced a prediction, CEM delivers dual insights through PP and PN analyses—revealing both the features that strongly support the current classification and those that would need to change to shift the prediction. This level of interpretability not only strengthens the clinical applicability of the model but also enables more informed decisions in complex diagnostic scenarios where biomarkers often interact non-linearly [28].

Furthermore, CEM aligns with the central objective of this study: to support early detection of ovarian cancer through an interpretable ML pipeline. Given that early-stage ovarian cancer is often asymptomatic and typically diagnosed at advanced stages, there is a growing emphasis on identifying subtle biomarker patterns that could inform earlier intervention. By leveraging CEM, we were able to reveal the specific combinations and shifts in laboratory parameters that most critically distinguish malignant from benign cases, thereby adding an interpretive layer to the model’s high accuracy performance.

In this study, the CEM, an XAI approach, was applied to a ML model that distinguishes ovarian and benign ovarian tumors. According to our findings, the decision-making mechanism of the model was transparently revealed thanks to the CEM and it was clearly understood which biomarkers were decisive in the classification process.

According to the results of the CEM analysis, in the analysis of a sample patient belonging to the “Cancer” class, it was observed that the model assigned the Cancer class with 99.68% accuracy; in this patient, it was found that LYM, RBC, PLT and PCT values strongly supported the classification. In the same patient, significant increases in age and HE4 values were determined as the most critical variables for class change.

Similarly, in the analysis conducted on a sample patient belonging to the “No Cancer” class, it was understood that the model classified with 99.74% accuracy and that the joint effect of multiple biomarkers was important rather than a single decisive biomarker in this class. However, PN analysis showed that decreases in hematological parameters such as RBC, LYM, PLT and CEA played a decisive role in the transition to Cancer class. In particular, the dramatic decrease observed in the RBC parameter stood out as the most critical change.

The findings generally show that the CEM enables us to understand not only the role of individual biomarkers but also how the interactions between these biomarkers are reflected in classification decisions. This lays a crucial foundation for developing multivariate, personalized decision-support systems that could aid in diagnosing early-stage ovarian cancer. CEM’s PP and PN analyses, which provide bidirectional explanations, reveal not only what the model bases its decisions on but also how specific parameters must vary for the decision to change. This enhances confidence in model decisions, particularly in high-risk clinical scenarios, and supports more active physician involvement in the process. In addition, to ensure the robustness of our findings, we also implemented relatively simpler machine learning models, including Lasso logistic regression and XGBoost. These models achieved comparable results to those obtained with the CEM framework, thereby reinforcing the consistency of the observed biomarker contributions. Furthermore, we applied the SHAP method, which offers a more basic level of explainability compared to CEM, and confirmed the alignment of its outcomes with those of the CEM analysis. This concordance across different modeling approaches underscores the reliability and generalizability of our results.

Despite the growing interest in XAI methods across various clinical domains, there remains a significant gap in the literature regarding the application of the CEM specifically in ovarian cancer diagnosis. While several studies have explored model interpretability using methods such as SHAP or LIME in oncology, few have addressed the utility of Contrastive Explanations that reveal both the supportive and counterfactual dimensions of model reasoning. To our knowledge, this is one of the first studies to apply CEM within a gynecologic oncology context, particularly for distinguishing malignant from benign ovarian tumors. This novelty highlights the importance of our findings and underlines the potential of CEM to inform biomarker-based decision-making in diseases where early detection is critical. The successful implementation of CEM in this study not only bridges a methodological gap but also introduces a new interpretive layer to clinical decision support systems for ovarian cancer.

This study demonstrates that the integration of the CEM into a ML-based diagnostic framework offers substantial value in the context of ovarian cancer classification. Our findings reveal that CEM not only highlights the contribution of individual biomarkers but also uncovers complex interactions between them that drive the model’s predictions. This multidimensional interpretability enables clinicians to understand why a patient is classified as “Cancer” or “No Cancer”, and more importantly, what minimal changes in biomarkers would reverse this classification. Such insight is critically important in diseases like ovarian cancer, where early detection is rare and prognosis is tightly linked to the stage at diagnosis.

By providing dual-sided explanations through PP and PN analyses, CEM empowers clinicians to move beyond static risk scores and engage with dynamic, patient-specific scenarios. These counterfactual insights align with the principles of personalized medicine, enabling more nuanced clinical decisions and potentially earlier interventions.

### Limitations

Despite the promising results, several limitations of this study should be acknowledged. First, the model was trained and evaluated on a retrospective, single-center dataset composed entirely of Chinese patients, which may limit the generalizability of the findings to other populations with different ethnic, genetic, or clinical backgrounds. Second, although the use of an open-access cohort enhances transparency and reproducibility, the absence of external validation on an independent dataset restricts the ability to confirm the model’s robustness and real-world applicability.

Third, while the Contrastive Explanation Method (CEM) provides improved interpretability and individualized insight into model decisions, further clinical validation in diverse healthcare settings is warranted to ensure its alignment with actual clinical reasoning processes. Future studies should therefore aim to replicate and externally validate the model in larger, multi-center, and multi-ethnic cohorts, ideally through prospective designs that incorporate clinical workflows and real-time diagnostics. Such efforts would improve confidence in both the predictive and interpretive utility of the proposed approach.

## 5. Conclusions

In summary, the integration of CEM into our analytical workflow was driven by its ability to generate counterfactual reasoning in a clinically meaningful way. Its capacity to dissect individual predictions and simulate plausible alternatives not only enhances model transparency but also offers potential for personalized diagnostic interpretation—making it an essential tool for the development of trustworthy artificial intelligence in gynecological oncology. The explainability and adaptability of CEM position it as a promising component in the next generation of clinical decision support systems, especially in cancers where early intervention dramatically affects survival.

## Figures and Tables

**Table 1:** Summary of Biomarker Levels and Statistical Differences Between Cancer and Non-Cancer Groups.

Variables	Cancer (*n* = 171)	No Cancer (*n* = 178)	*p* *
	Median (Min–Max)	Median (Min–Max)	
AFP	2.44 (0.61–1210)	2.1 (0.61–20.53)	0.013
Age	53 (18–83)	36 (15–69)	<0.001
ALB	39.2 (22–51.5)	43.4 (28–50.9)	<0.001
ALP	77 (26–763)	65 (29–157)	<0.001
CA125	241.5 (7.26–5000)	22.66 (3.75–515.4)	<0.001
CEA	1.41 (0.2–138.8)	1.27 (0.2–6.95)	0.103
HE4	140.9 (29.49–3537.6)	43.77 (16.71–531.8)	<0.001
LYM	1.34 (0.35–3.2)	1.67 (0.38–3.49)	<0.001
PCT	0.26 (0.09–0.69)	0.23 (0.07–0.42)	<0.001
PLT	265 (74–868)	223.5 (88–398)	<0.001
RBC	4.32 (2.62–6.74)	4.42 (2.9–5.39)	0.031
TBil	7.6 (2.5–22.7)	9.2 (2.7–38.3)	<0.001

* Mann–Whitney U test, Min: Minimum, Max: Maximum.

**Table 2:** Performance Evaluation of the Deep Neural Network Model in Classifying Ovarian Cancer.

Performance Metrics	Value	95% CI
Accuracy	0.950	0.901–0.975
Balanced Accuracy	0.950	0.914–0.986
F1-Score	0.955	0.904–1.000
Matthews’ correlation Coefficient	0.899	0.811–0.986
Sensitivity	0.949	0.879–0.978
Specificity	0.951	0.869–0.980
Youden’s index	0.900	0.828–0.973
Positive Predictive Value	0.962	0.895–0.985
Negative Predictive Value	0.935	0.849–0.971

**Table 3:** Classification Performance Metrics Across Different Thresholds.

Threshold	Context	Sens	Spec	PPV	NPV	F1	Acc	Bal Acc	MCC	Youden
0.300	High Sensitivity	0.951	0.949	0.935	0.962	0.943	0.950	0.950	0.899	0.900
0.500	Default	0.951	0.949	0.935	0.962	0.943	0.950	0.950	0.899	0.900
0.100	Optimal (Youden’s J)	0.967	0.949	0.937	0.974	0.952	0.957	0.958	0.914	0.917
0.700	High Specificity	0.934	0.949	0.934	0.949	0.934	0.943	0.942	0.884	0.884
0.900	High Specificity	0.885	0.949	0.931	0.915	0.908	0.921	0.917	0.840	0.835

**Table 4:** Contrastive Explanation Method (CEM) Analysis for a Patient Diagnosed with Ovarian Cancer.

Variables	Original Value	PN Value	Change	PP Value	Comment
AFP	−0.11731216	−0.11731216	→	−9.28 × 10^−10^	Minimal contribution to change
Age	−0.13393328	0.23358673	↑↑	−1.94 × 10^−9^	Critical: Significant increase required for transition to “No Cancer” class
ALB	0.21455381	0.21455382	→	−4.46 × 10^−9^	Minimal contribution to change
ALP	−0.21772664	−0.21772663	→	−3.48 × 10^−9^	Minimal contribution to change
CA125	−0.44230699	−0.442307	→	1.08 × 10^−9^	Minimal contribution to change
CEA	−0.22786435	−0.22786435	→	6.98 × 10^−10^	Minimal contribution to change
HE4	−0.42683999	−0.0499623	↑↑	1.26× 10^−8^	Critical: Significant increase required for transition to “No Cancer” class
LYM	1.07689711	1.0768971	→	1.35734686	Important: Strong positive contribution to “Cancer” classification
PCT	1.30060536	1.3006054	→	3.60143817 × 10^−2^	Important: Positive contribution to “Cancer” classification
PLT	0.65023627	0.65023625	→	3.84033675 × 10^−1^	Important: Positive contribution to “Cancer” classification
RBC	0.77667959	0.7766796	→	1.17899583	Important: Strong positive contribution to “Cancer” classification
TBil	−0.27157329	−0.27157328	→	−1.06535471 × 10^−8^	Minimal contribution to change

→: Minimal contribution to change or important contribution; ↑↑: Critical contribution.

**Table 5:** Contrastive Explanation Method (CEM) Analysis for a Patient Without Ovarian Cancer.

Variables	Original Value	PN Value	Change	PP Value	Comment
AFP	−0.09594891	−0.21987575	↓	−1.62 × 10^−9^	Important: Decrease required for transition to cancer class
Age	0.19524076	0.19524077	→	−1.58 × 10^−9^	No contribution to change
ALB	−0.32864775	−0.32864776	→	1.26 × 10^−8^	Supportive of normal diagnosis
ALP	−0.32042685	−0.5816372	↓	2.03 × 10^−9^	Important: Decrease required for class change
CA125	−0.04608445	−0.04608445	→	−2.77 × 10^−10^	Supportive of normal diagnosis
CEA	−0.21545296	−0.37999222	↓	−7.16 × 10^−9^	Important: Decrease required for class change
HE4	0.11000195	0.11000195	→	−2.62 × 10^−9^	No contribution to change
LYM	−0.56240115	−0.9324508	↓	2.31 × 10^−8^	Important: Decrease required for class change
PCT	−0.59422663	−0.59422666	→	2.91 × 10^−8^	Supportive of normal diagnosis
PLT	−0.94900587	−1.067009	↓	−2.32 × 10^−8^	Important: Decrease required for transition to cancer class
RBC	0.08232532	−0.9617363	↓↓	−6.71 × 10^−10^	Critical: The parameter requiring the most significant change
TBil	0.89335305	0.89335304	→	2.559 × 10^−9^	Minimal contribution to change

→: Minimal contribution to change or important contribution; ↓, ↓↓: Critical contribution.

**Table 6:** Comparison of performance metrics for models.

Metrics	Lasso Logistic Regression	XGBoost	Deep Neural Network
Accuracy	0.9500	0.9500	0.95
AUC	0.9638	0.9751	0.961
Sensitivity	0.9722	0.9583	0.955
Specificity	0.9265	0.9412	0.899
PPV	0.9333	0.9452	0.949
NPV	0.9692	0.9552	0.951
F1-Score	0.9524	0.9517	0.955

**Table 7:** Feature Importance Scores Derived from LASSO-Logistic Regression and XGBoost Models.

Feature	Random Forest Importance	XGBoost Importance
HE4	0.251411	0.383316
Age	0.185992	0.068233
CA125	0.154372	0.082904
CEA	0.056677	0.098949
ALB	0.084424	0.067419
LYM	0.055859	0.052107
PCT	0.038255	0.052409
PLT	0.045608	0.045485
AFP	0.031367	0.043531
RBC	0.025394	0.040433
ALP	0.038658	0.036015
TBIL	0.034025	0.030098

## Data Availability

The data used in this study were obtained from a publicly available dataset reported in a previously published study: ref. [15]. The dataset can be accessed at ref. [15].
